# Detection of *Campylobacter jejuni* in Lizard Faeces from Central Australia Using Quantitative PCR

**DOI:** 10.3390/pathogens6010001

**Published:** 2016-12-23

**Authors:** Harriet Whiley, Ryan McLean, Kirstin Ross

**Affiliations:** Health and the Environment, School of the Environment, Flinders University, GPO BOX 2100, Adelaide 5001, Australia; ryansmclean@gmail.com (R.M.); Kirstin.Ross@flinders.edu.au (K.R.)

**Keywords:** *Campylobacter jejuni*, *Campylobacter* spp., campylobacteriosis, lizard, environmental reservoir, public health, reptile

## Abstract

Worldwide, *Campylobacter* is a significant cause of gastrointestinal illness. It is predominately considered a foodborne pathogen, with human exposure via non-food transmission routes generally overlooked. Current literature has been exploring environmental reservoirs of campylobacteriosis including potential wildlife reservoirs. Given the close proximity between lizards and human habitats in Central Australia, this study examined the presence of *Campylobacter jejuni* from lizard faeces collected from this region. Of the 51 samples collected, 17 (33%) (this included 14/46 (30%) wild and 3/5 (60%) captive lizard samples) were positive for *C. jejuni* using quantitative PCR (qPCR). This was the first study to investigate the presence of *C. jejuni* in Australian lizards. This has public health implications regarding the risk of campylobacteriosis from handling of pet reptiles and through cross-contamination or contact with wild lizard faeces. Additionally this has implication for horizontal transmission via lizards of *C. jejuni* to food production farms. Further research is needed on this environmental reservoir and potential transmission routes to reduce the risk to public health.

## 1. Introduction

*Campylobacter jejuni* is a pathogen of significant public health concern [1,2,3]. It is one of the main causative agents of campylobacteriosis, which worldwide is a common gastrointestinal disease [2,4,5]. Campylobacteriosis is generally self-limiting; however, more severe cases may require medical attention and infection has been linked to other complications including Guillain-Barré syndrome, reactive arthritis, and irritable bowel syndrome [2]. Over the past decade there has been a significant increase in the incidence of campylobacteriosis in developed and developing countries [1]. It has been estimated that annually there are 9.2 million cases of campylobacteriosis in the European Union [6] and 1 million cases in the USA [7]. In Australia in 2015 there were a total of 22,564 notified cases of campylobacteriosis, with the true incidence likely to be significantly higher due to many cases going undiagnosed [8,9]. Typically, campylobacteriosis is considered a foodborne illness; however, there has been increasing evidence to suggest that environmental reservoirs may also play a significant role in disease transmission [3,4,5]. Wildlife are considered both potential infectious reservoirs and a source or mechanism enabling cross-contamination of surface waters and other environments such as poultry and produce farms [10,11,12].

Previous research has suggested that lizards play a role in the spread of human bacterial pathogens [13,14]. However, this has predominately been focused on *Salmonella* spp. [15,16,17,18,19,20,21,22,23,24,25] or *Escherichia coli* [22,26]. Gilbert et al. [27] investigated the presence and host association of intestinal *Campylobacter* spp. in reptiles from captive populations in Europe and found that 63/163 (38%) of lizards were positive using PCR detection and 18/163 (11%) were positive using culture. Additionally, a genetically distinct variant of *Campylobacter fetus* has been isolated in the USA from both reptiles and humans who had direct or indirect contract with the reptile [28]. This is also supported by a study from Taiwan that detected *C. fetus* from 6.7% (12/179) of faeces collected from wild and domestic reptiles [29].

Lizards are a widespread group of squamate reptiles [30] that can be found across Australia and have adapted to the wide range of environments [31]. Their ability to inhabit diverse environments is one reason that lizards are commonly found living close to human habitation [32]. This is the first study to use quantitative PCR (qPCR) to investigate the presence of *C. jejuni* in lizard faeces (scats) collected from across Central Australia. The incidence of *C. jejuni* in relation to the proximity to human habitation was also explored.

## 2. Results

**Figure 1:**
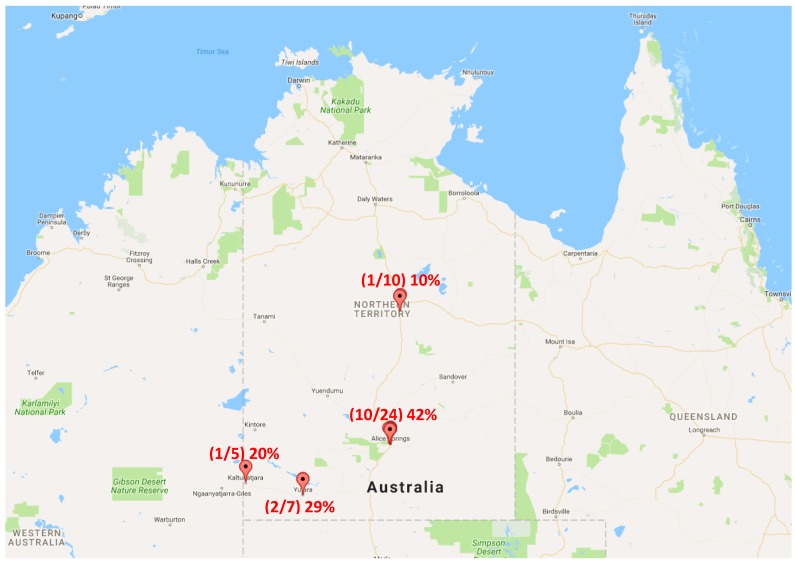
Sampling locations and the percentage of lizard scats that were positive for *Campylobacter jejuni* using quantitative PCR (this excludes captive lizards) [33].

Of the 51 samples collected around Central Australia, 17 (33%) (this included 14/46 (30%) wild and 3/5 (60%) captive lizard samples) were positive for *C. jejuni* using qPCR. The highest proportion of positive samples was found around Alice Springs with 10/24 (42%) samples returning a positive result for *C. jejuni*. Next, 2/7 (29%) of the samples collected from the Yulara community, 1/5 (20%) samples from the Kaltukatjara community and 1/10 (10%) from Tenant Creek were positive for *C. jejuni* (Figure 1). Three of the five scats collected from captive lizard were positive for *C. jejuni*.

## 3. Discussion

This was the first study to investigate the presence of *C. jejuni* in Australian lizards. The presence of *C. jejuni* DNA in both wild and captive lizards has public health significance. It is important to note that the detection method used was qPCR and as such both viable and killed *Campylobacter* would have been detected. However, one of the benefits of qPCR over culture is that it can detect viable but non-culturable organisms [34]. Future studies are needed to obtain isolates using culture as these provide opportunity for further analysis or examination of isolate diversity and phenotypic characteristics [27]. Another limitation of this study is the lack of lizard speciation; as such further research is required to characterize the epidemiology and ecology in *C. jejuni* in lizard populations.

Previous studies have identified that domestic reptiles being kept as pets may be associated with the spread of zoonotic diseases including salmonellosis, mycobacteriosis, chlamydophilosis, *Aeromonas* and *Pseudomonas* infections [35,36]. The results from this study suggest that campylobacteriosis should also be considered as a potential zoonotic disease that may be spread through the handling of lizards. It is important that new reptile owners are educated about the risks associated with handling lizards and appropriate ways to protect themselves [35].

The presence of *C. jejuni* in 30% of wild lizard faeces demonstrates that the bacterium is quite common in the lizard population found in Central Australia. This has public health implications as contact with the contaminated faeces may occur directly or indirectly through cross-contamination of other surfaces. This is particularly important as the dose rated for campylobacteriosis has been shown to be as low as 800 colony-forming units (CFU) [37]. Additionally, although *Campylobacter* cannot typically replicate outside of a host it can survive in the environment, with the survival time depending on numerous variables such as temperature, light, moisture and nutrient content [3]. There have been several studies investigating the survival of *C. jejuni* in bovine faeces, with the survival time ranging from 1.2 days to 32 days [38,39]. However, there have been limited studies on the survival of *C. jejuni* in lizard faeces.

The presence of *C. jejuni* in wild lizard species also has implications for horizontal transmission to food production farms. Previous studies have demonstrated *Campylobacter* can be spread to broiler farms through vectors including flies [40] beetles [41] and rodents [42]. However there has been limited research into the spread of campylobacteriosis via lizards and other reptiles. Further research is needed to identify the significance of *C. jejuni* contaminated lizard faeces and the implications for food production farms.

## 4. Materials and Methods

### 4.1. Sample Collection

A total of 51 lizard faecal samples (scats) were collected from locations across Central Australia (Figure 1) including towns, remote communities and tourist areas. Of these, 46 were from unknown lizards and five were collected from captive lizards including four *Pogona vitticeps* (bearded dragons) and a *Rhynchoedura ornate* (Western beaked gecko). Sampling was by convenience and information regarding the location of each sample, surface type it was collected from and surrounding habitat description was recorded. Samples were identified as reptile faeces differ from those of mammals. Mammals excrete both the faecal pellet and urine through separate openings while reptiles have a single opening for both and consequently they are often excreted together [43]. Reptiles produce uric acid instead of urine and this is then deposited and excreted in the same way as the digestive waste, resulting in a very distinct faecal pellet [44].

Faecal samples were collected using sterile tweezers and deposited into sterile Eppendorf tubes. Collected samples were stored with silica gel beads (>4 grams per gram of faeces) and stored at between 2 °C and 5 °C in an opaque storage container [45], to limit further degradation by light or temperature fluctuation until the DNA was extracted.

Ethics approval (approval number 6464) for this project was given by the Social and Behavioural Research Ethics Committee (SBREC) and the Low Risk Sub-Committee at Flinders University as meeting the National Statement on Ethical Conduct in Human Research (March 2007).

### 4.2. qPCR Detection of Campylobacter jejuni

Faecal samples were aseptically crushed and mixed using a sterile mortar and pestle. Approximately 0.05 g was used for the DNA extraction which was done using the FastDNA^®^ Spin Kit for Soil (MP Biomedicals, Santa Ana, CA, USA) following manufacturer’s instructions. The FastDNA^®^ Spin Kit for Soil was chosen as it has been shown to effectively extract DNA from faecal samples, producing greater yields than other commercially available kits [46].

Duplicate qPCR of both the neat and a 1/10 dilution of the DNA extract was then performed to detect the presence of *C. jejuni*. The 1/10 dilutions of the DNA extracts were performed to account for the possible presence of PCR inhibitors. *C. jejuni* qPCR was performed using previously described primers and probe which targets the *C. jejuni* gene *mapA* (X80135) [34,47]. Best et al. [47] demonstrated there was no amplification for unrelated organisms such as *E. coli, Helicobacter pylori, Campylobacter lari, Campylobacter upsaliensis, Campylobacter curvus, Campylobacter helveticus* and *C. fetus*, and only 0.1% of samples were positive for both *C. jejuni* and *E. coli.* Briefly, the 20 µL reaction volume contained 1× SsoAdvanced universal probes supermix (Bio-Rad, Gladesville, NSW, Australia), 300 nM forward primer: 5′-CTGGTGGTTTTGAAGCAAAGATT-3′; 300 nM reverse primer: 5′-CAATACCAGTGTCTAAAGTGCGTTTAT-3′; 100 nM probe: 5′-FAM TTGAATTCCAACATCGCTAATGTATAAAAGCCCTTT-3′ TAMRA and 5 µL of sample DNA. The cycling conditions included an initial hold at 95 °C for 3 min, followed by 50 cycles consisting of 95 °C for 15 s, 60 °C for 30 s. All PCR runs contained a positive *C. jejuni* control and a non-template control.

## 5. Conclusions

Campylobacteriosis is typically considered a foodborne illness; however, increasingly research is demonstrating the importance of environmental reservoirs. This was the first study to detect *C. jejuni* from wild and captive lizard faeces collected across Central Australia. Using qPCR, 14/46 of the wild lizard faeces and 3/5 of the captive lizard faeces were positive for *C. jejuni*. This has public health implications regarding the risk of campylobacteriosis from handling of pet reptiles and through cross-contamination or contact with wild lizard faeces. Additionally this has implication for horizontal transmission via lizards of *C. jejuni* to food production farms. Further research is needed on this environmental reservoir and potential mechanisms to reduce the risk to public health.
